# Hospitalisation with Infection, Asthma and Allergy in Kawasaki Disease Patients and Their Families: Genealogical Analysis Using Linked Population Data

**DOI:** 10.1371/journal.pone.0028004

**Published:** 2011-11-28

**Authors:** Rebecca J. Webster, Kim W. Carter, Nicole M. Warrington, Angeline M. Loh, Sophie Zaloumis, Taco W. Kuijpers, Lyle J. Palmer, David P. Burgner

**Affiliations:** 1 Centre for Genetic Epidemiology and Biostatistics, University of Western Australia, Perth, Australia; 2 Division of Bioinformatics and Biostatistics, Telethon Institute for Child Health Research, UWA Centre for Child Health Research, University of Western Australia, Perth, Australia; 3 School of Women's and Infants' Health, University of Western Australia, Perth, Australia; 4 Centre for Molecular, Environmental, Genetic and Analytic Epidemiology, University of Melbourne, Melbourne, Australia; 5 Department of Pediatric Hematology, Immunology and Infectious Diseases, Emma Children's Hospital, Academic Medical Center (AMC), Amsterdam, The Netherlands; 6 School of Pediatrics and Child Health, University of Western Australia, Perth, Australia; 7 Murdoch Childrens Research Institute, Royal Children's Hospital, Parkville, Australia; Universite de Montreal, Canada

## Abstract

**Background:**

Kawasaki disease results from an abnormal immunological response to one or more infectious triggers. We hypothesised that heritable differences in immune responses in Kawasaki disease-affected children and their families would result in different epidemiological patterns of other immune-related conditions. We investigated whether hospitalisation for infection and asthma/allergy were different in Kawasaki disease-affected children and their relatives.

**Methods/Major Findings:**

We used Western Australian population-linked health data from live births (1970–2006) to compare patterns of hospital admissions in Kawasaki disease cases, age- and sex-matched controls, and their relatives. There were 295 Kawasaki disease cases and 598 age- and sex-matched controls, with 1,636 and 3,780 relatives, respectively. Compared to controls, cases were more likely to have been admitted at least once with an infection (cases, 150 admissions (50.8%) vs controls, 210 admissions (35.1%); odds ratio (OR) = 1.9, 95% confidence interval (CI) 1.4–2.6, *P* = 7.2×10^−6^), and with asthma/allergy (cases, 49 admissions (16.6%) vs controls, 42 admissions (7.0%); OR = 2.6, 95% CI 1.7–4.2, *P* = 1.3×10^−5^). Cases also had more admissions per person with infection (cases, median 2 admissions, 95% CI 1–5, vs controls, median 1 admission, 95% CI 1–4, *P = *1.09×10^−5^). The risk of admission with infection was higher in the first degree relatives of Kawasaki disease cases compared to those of controls, but the differences were not significant.

**Conclusion:**

Differences in the immune phenotype of children who develop Kawasaki disease may influence the severity of other immune-related conditions, with some similar patterns observed in relatives. These data suggest the influence of shared heritable factors in these families.

## Introduction

Kawasaki disease (KD) is an acute febrile vasculitis of unknown aetiology that predominantly affects infants and pre-school children. Probably uniquely amongst childhood illnesses, KD damages the coronary arteries and is the leading cause of pediatric acquired heart disease in developed countries.[1], [2] The consensus is that KD is triggered by to one (or more) widely distributed but as yet unidentified infectious agent(s) that precipitate an abnormal immunological response in genetically susceptible individuals.[2], [3]

There is a significant genetic contribution to KD susceptibility.[4] Epidemiological data indicate that KD segregates in families, with the parents of children with KD significantly more likely to have had the disease themselves in childhood.[5] The heritability (λ_s_) of KD in the Japanese is 6–10,[6] and a similar value is suggested in Koreans (Burgner D, unpublished observations); no analogous data are reported from other populations. The incidence of KD in Japanese Americans born in Hawaii is higher than that reported in Japan and over 10 times greater than that in US Caucasians.[7] Genomic data, both from candidate gene and more recently from genome-wide linkage[8] and association analyses,[9], [10], [11] have identified variants, particularly in immune- and cardiovascular-related genes, that may contribute to KD susceptibility.

We hypothesised that heritable differences in immune responses in Kawasaki disease would influence the frequency and severity of other immune-related conditions. We investigated whether children with KD and their relatives had different epidemiological patterns of infectious diseases, asthma/allergy and autoimmune diseases using total linked population data, with hospital admission as a measure of disease severity.

## Methods

### Objectives

Given the likely immunogenetic contribution to KD susceptibility, we hypothesised that diseases for which immune function is important in determining susceptibility and severity might show distinct epidemiological patterns in KD-affected children and their biological relatives.

### Setting

Western Australia (WA, total population ∼2.29 million) has a unique resource of linked population health data (The WA Data Linkage System, WADLS), which integrates complete population health (including hospital admissions) and other key demographic data from 1969 onwards.[12] The WA Family Connections Genealogical Project has used this resource to develop a system of linkages within WALDS representing genealogical relationships for residents of WA.[13] We used these resources to compare the epidemiology of hospital admissions with infectious diseases, asthma/allergy, and autoimmune diseases in all KD cases diagnosed in Western Australia between 1974 and 2006, and their relatives, with data from matched non-KD children and their families.

### Participants

We analysed data from WADLS, which comprises linked data from statutory collections, including the WA Registry of Births, Deaths and Marriages, and the Hospital Admissions Data Collection (HMDC), from 1970 onwards.[14] These datasets have been extensively validated previously.[12], [15], [16] A cohort of KD cases and controls was selected from the WADLS by firstly identifying individuals whose births were recorded in the WA Birth Registry between 1974 and 2006. From this group, all available KD cases were selected using hospital admission records (from the HMDC), using a discharge diagnostic coding of KD (the International Classification of Diseases (ICD) version 10 code, M30.3, or the ICD version 9 code, 446.1). Two siblings with KD were identified; one was excluded from analyses to maintain an unrelated case population. Age- and sex-matched controls for the KD cases were selected from those individuals with neither a diagnosis of KD nor a history of KD in their 1^st^, 2^nd^ or 3^rd^ degree relatives, according to the HMDC, with a ratio of approximately two controls to each KD case. All cases and controls were unrelated to each other to within three degrees. Genealogical information for cases and controls, available through the linked Family Connections Project database,[13] was used to identify all 1^st^, 2^nd^ and 3^rd^ degree relatives of cases and controls. In total 1,636 relatives of KD cases and 3,780 relatives of controls were identified. Admission to hospital was used as a marker of infection severity.[17], [18] Birth, death and hospital admission data for each individual were extracted from subsets of the Birth Registry (1974–2006), Death Registry (1970–2006) and HMDC (1970–2006) databases. Data on Aboriginality and self-determined ethnicity were not readily available. Individuals born outside of WA, and therefore not recorded on the Birth Registry, were not included.

### Classification of hospital admissions

We initially analysed the data with respect to seven disease groups; cardiovascular disease, asthma/allergy, autoimmune diseases, infectious diseases, Crohn disease, ulcerative colitis and psoriasis. The ICD codes used to define each of the broad disease groups were defined *a priori* (Supplementary Table S1). ICD codes for infectious disease admissions were grouped into clinically meaningful categories using the Clinical Classification Software (CCS).[19] All ICD codes for hospital admissions were standardised to ICD Version 10.5 codes prior to analysis. We calculated the incidence of each disease group (censored after the first admission) and the total number of admissions per individual in each of the four study populations (KD cases, non-KD controls, KD case relatives, non-KD control relatives). The numbers of admissions with Crohn disease, psoriasis and ulcerative colitis were too low to permit further analysis. Analysis of cardiovascular disease admissions was restricted to relatives' data only, as cardiovascular disease admissions in cases and controls were only related to investigation of cardiovascular sequelae of KD.

### Statistical methods

Hospital admission data for infectious diseases, asthma/allergy, and autoimmune diseases in KD cases and controls were initially analysed by comparing the incidence of diagnoses in each disease group between cases and controls, using a Monte Carlo method for sparse data implemented in the CLUMP software v2.3.[20] The Mann-Whitney test was used to compare the age at admission and the number of admissions per person for each disease group in KD cases and controls.

The hospital admission data for the relatives of KD cases and controls were analysed using generalised linear mixed models (GLMMs) to model the effects of covariates on disease-related variables and therefore account for the correlation between related individuals. The correlation between outcomes from related individuals must be accounted for in order to obtain appropriate inferences about the fixed effects (or exposures); the standard errors of the fixed effect estimates will be incorrect if the correlation is not modelled. The GLMMs analyses utilised the glmmPQL function in the statistical analysis package, R v2.11.0,[21] and accounted for family groups by using the family ID to describe a contribution to the random effects. The incidence of each disease was analysed as a binomial outcome, while the age-at-onset and number of admissions per person were analysed as continuous outcomes. Age-at-onset outcomes were analysed under a Gaussian model, with the asthma/allergy, and infectious disease outcomes being log-transformed prior to analysis. The numbers of admissions per person for all diseases were analysed under a Poisson model. For each model, a covariate was included to adjust for the relationship of an individual to a KD case. Additional covariates for each model were selected by initially including both (where appropriate) of the covariates sex and age in the analysis, and removing those not significantly associated with the outcome in a step-wise manner. Using a binary covariate indicating the presence or absence of a relative of any degree (1^st^, 2^nd^ or 3^rd^) with KD, analyses were performed on the population of all relatives of KD cases and controls, and on male and female relatives separately. Using a factored covariate indicating the degree of relatedness of an individual to a KD case (0, 1, 2 or 3), analyses were performed on a subset of the population of all relatives. This subset included all 3,780 control relatives plus a reduced set of 1,228 KD case relatives, obtained by excluding the pedigrees of cases with incomplete or inconsistent family data. With this subset we were able to calculate the degree of relatedness between cases and their relatives using R's Kinship package (http://cran.r-project.org/web/packages/kinship/index.html).

The incidence of diseases in the relatives of KD cases was also investigated by calculating the relative risk (RR) of each of the disease groups in the relatives of KD cases compared to the relatives of controls, using a random-effects Cox proportional hazards model. These analyses were performed on a subset of 1,094 KD case relatives and 2,232 control relatives from pedigrees satisfying all of the criteria of the *familycheck* function in R's Kinship package. Analyses were performed using the *coxme* function of the Kinship package. The number of 3^rd^ degree relatives of KD cases born in WA (and therefore on whom childhood admission data were available) was too small to permit detailed analysis, which was therefore restricted to 1^st^ and 2^nd^ degree relatives of KD cases and controls.

### Ethics

This study was approved by the University of Western Australia Human Research Ethics Committee and the Western Australian Department of Health Human Research Ethics Committee.

## Results

### Infectious disease hospital admissions

**Figure 1:**
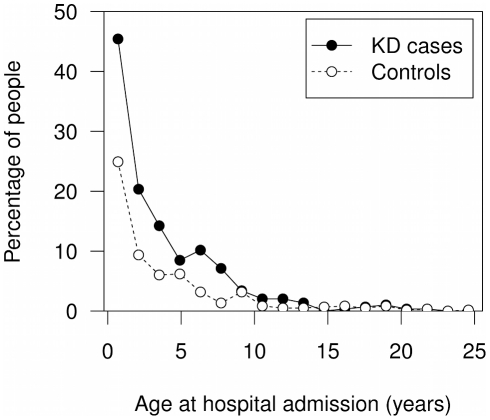
Age at hospital admission with infectious diseases in KD cases and controls. KD case admissions coded as infectious disease up to 2 weeks prior to KD diagnosis have been excluded.

There were 295 KD cases and 598 non-KD age- and sex-matched controls, with 1,636 and 3,780 relatives, respectively. The median age of KD diagnosis was 3 years (95% confidence interval (CI) 0–9 years). The KD case group had a higher proportion of individuals with at least one infectious disease admission (150/295, 50.8%) than the control group (210/598, 35.1%); odds ratio (OR) = 1.9, 95% CI 1.4–2.6, *P* = 7.2×10^−6^ (Table 1, Supplementary Figure S1). The number of infectious disease admissions per person was also higher in KD cases (median of 2 admissions, 95% CI 1–5) than in controls (median of 1 admission, 95% CI 1–4), *P = *1.09×10^−5^ (Table 1). Most infectious disease admissions occurred prior to 10 years of age in KD cases and controls (Figure 1). The majority of infectious disease admissions in cases (94% of total) occurred prior to the diagnosis of KD. To account for the possibility that infectious disease admissions in KD cases may have been inflated by admissions during the KD illness but prior to KD diagnosis, 19 infectious disease admissions occurring two weeks prior to KD admission were excluded. The proportion of individuals admitted at least once with an infectious disease remained significantly higher in KD cases than in controls (OR = 1.5, 95% CI 1.1–2.0, *P* = 0.01) (Table 1). The number of admissions per person for infectious diseases also remained significantly higher in KD cases following adjustment for the two weeks prior admissions (*P* = 5.50×10^−6^) (Table 2, Figure 1). There was no significant difference between KD cases and controls in the age of the first hospital admission with any diagnostic grouping (Table 1).

**Table 1:** Population descriptives and comparison of the incidence of diseases in KD cases and controls and their relatives.

	KD cases, *n*(%)	Controls, *n*(%)	Odds ratio (95% CI)	Chi-square *P* value	Relatives of KD cases, *n*(%)	Relatives of controls, *n*(%)
Cohort	295 (100%)	598 (100%)			1636 (100%)	3780 (100%)
Male	183 (62.0%)	376 (62.9%)			834 (51.0%)	1937 (51.2%)
KD	295 (100%)	0 (0%)			0 (0%)	0 (0%)
Infectious diseases	150 (50.8%)	210 (35.1%)	1.9 (1.4, 2.6)	7.2×10^−6 ‡^	561 (34.3%)	1379 (36.5%)
Infectious diseases (adjusted)*	131 (44.4%)	210 (35.1%)	1.5 (1.1, 2.0)	0.01 †	-	-
Asthma/allergy	49 (16.6%)	42 (7.0%)	2.6 (1.7, 4.2)	1.3×10^−5 ‡^	151 (9.2%)	332 (8.8%)
Autoimmune diseases	8 (2.7%)	7 (1.2%)	2.4 (0.7, 7.7)	0.10	37 (2.3%)	70 (1.9%)

† *P*<0.05; ^‡^ *P*<0.001; * admissions ≤2 weeks prior to KD diagnosis excluded.

**Table 2:** Comparison of age at onset and number of hospital admissions per person, of diseases in KD cases (*n* = 295) and controls (*n* = 598).

	Age at onset			Hospital admissions per person		
	KD cases, median years (95% CI)	Controls, median years (95% CI)	Mann-Whitney *P* value	KD cases, median years (95% CI)	Controls, median years (95% CI)	Mann-Whitney *P* value
Infectious diseases	1 (0, 8)	1 (0, 12)	0.31	2 (1, 5)	1 (1, 4)	1.09×10^−5^ ‡
Infectious diseases (adjusted)*	1 (0, 9)	1 (0, 12)	0.38	2 (1, 5)	1 (1, 4)	5.50×10^−6^ ‡
Asthma/allergy	2 (0, 8)	2 (0, 14)	0.46	1 (1, 4.6)	1 (1, 4)	0.81
Autoimmune diseases	4.5 (0.4, 8.6)	9 (1.9, 15.7)	0.12	1 (1, 1)	1 (1, 3.8)	0.35

‡ *P*<0.001; *admissions ≤2 weeks prior to KD diagnosis excluded.

The frequencies of clinical diagnostic categories of infectious diseases showed that admission with clinically diagnosed ‘viral infection’ (CCS code 7) was significantly more common in KD cases (61 of 434 total infectious disease admissions, 14.1%) than in non-KD controls (35/463, 7.6%), *P*<1×10^−6^ (Supplementary Table S2). ‘Skin and subcutaneous infection’ (CCS code 21) admissions were less common in KD cases (21, 4.8%) than controls (35, 7.6%), although the differences were not significant. Similar patterns of infectious disease admissions (i.e. increased viral infections, decreased skin and subcutaneous infections) were observed in the relatives of KD cases compared to the non-KD relative groups, although differences were not significant. Other clinical infection categories were largely similar in KD cases and controls and their relatives (Supplementary Table S2).

### Asthma/allergy hospital admissions

**Figure 2:**
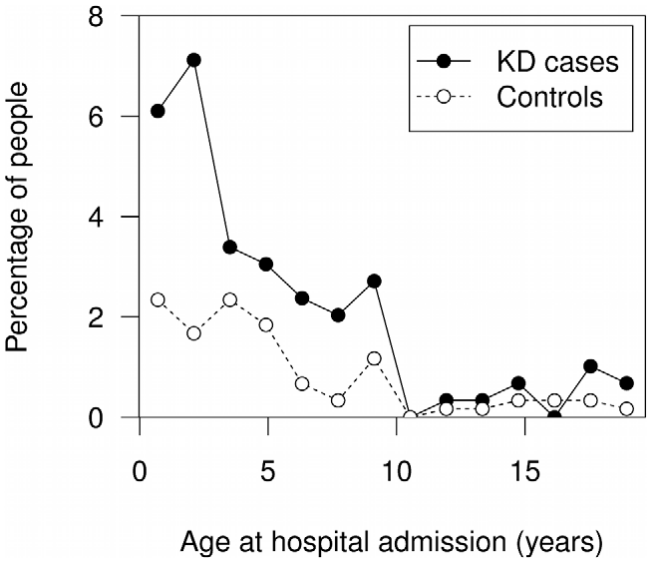
Age at hospital admission with asthma/allergy in KD cases and controls.

KD cases were more likely to have been admitted at least once with asthma/allergy (KD cases 49 admissions (16.6%) *vs* controls 42 admissions (7.0%); OR = 2.6, 95% CI 1.7–4.2, *P* = 1.3×10^−5^ (Table 2). Asthma accounted for 57 of the 90 admissions (63.3% of total), and allergic disease for the remainder (Supplementary Table S3). The majority of admissions for asthma/allergy occurred before the age of 10 years (Figure 2). The number of admissions per person for asthma/allergy did not significantly differ between KD cases and controls (Table 2).

The generalized linear mixed models (GLMM) analysis showed no significant overall differences between the relatives of the KD cases and controls in incidence, age at onset or number of admissions per person for any disease group (Table 3). No significant associations were observed when analyses were stratified by gender (data not shown). Second degree relatives of KD cases had more admissions per person with asthma/allergy (Coefficient  = 1.11, 95% CI 0.73–1.49, *P*<0.0001) (Table 4). The results of random-effects Cox proportional hazards model analyses of disease incidence in case and control relatives were consistent with GLMM results; the risk of hospital admission for infectious disease was approximately three-fold higher in 1^st^ degree relatives of KD cases compared to 1^st^ degree relatives of controls, although this was not statistically significant (data not shown).

**Table 3:** Generalized linear mixed model analyses of the incidence, age at onset, and number of hospital admissions per person of diseases in relatives of KD cases and controls, clustering by family.

	Incidence		Age at onset (years)		Hospital admissions per person	
	OR (95% CI)	*P* value	Coefficient (95% CI)	*P* value	Coefficient (95% CI)	*P* value
Asthma/allergy§	1.11 (1.11, 0.12)	0.35	1.12 (−1.16, 1.44)	0.40	0.13 (−0.09, 0.34)	0.25
Autoimmune diseases^#^	1.23 (1.22, 1.23)	0.38	4.87 (4.26, 5.47)	0.13	−0.21 (−0.48, 0.05)	0.12
Infectious diseases§	1.06 (1.05, 1.06)	0.49	−1.01 (−1.17, 1.15)	0.91	0.02 (−0.10, 0.13)	0.76

Results given are for a binary term indicating the presence or absence of a relative with KD.

§ The asthma/allergy, and infectious disease age at onset outcomes were adjusted for sex; ^#^ The autoimmune disease incidence outcome was adjusted for sex and age in 2008. The autoimmune disease number of hospital admissions per person outcome was adjusted for age in 2008.

**Table 4:** Results of generalized linear mixed model analyses of the incidence, age at onset and number of hospital admissions per person, of diseases in relatives of KD cases (*n* = 1,636) and controls (*n* = 3,780), clustering by family, and stratified by degree of relatedness to an individual with KD, with data from the relatives of controls acting as the baseline.

			Incidence		Age at onset (years)		Hospital admissions per person	
	Factor	*n* (%)	OR (95% CI)	*P*	Coefficient (95% CI)	*P*	Coefficient (95% CI)	*P*
CVD*	Relatives of controls	59 (1.6%)			Median = 47		Median = 1	
	1° relatives of cases	11 (1.3%)	0.79 (0.79, 0.80)	0.50	−0.45 (−1.26, 0.37)	0.90	−0.07 (−0.63, 0.49)	0.81
	2° relatives of cases	5 (2.7%)	1.54 (1.52, 1.57)	0.43	−2.92 (−4.08, −1.76)	0.57	−0.34 (−1.24, 0.57)	0.47
	3° relatives of cases	0 (0%)	2.46×10^−9^ (0,∞)	1	-	-	-	-
Asthma/allergy^§^	Relatives of controls	332 (8.8%)			Median = 7		Median = 1	
	1° relatives of cases	69 (8.1%)	0.99 (0.98, 0.99)	0.92	1.10 (−1.28, 1.55)	0.59	−0.10 (−0.37, 0.16)	0.45
	2° relatives of cases	16 (8.6%)	1.09 (1.09, 1.10)	0.73	1.41 (−1.37, 2.72)	0.31	1.11 (0.73, 1.49)	<0.001^†^
	3° relatives of cases	2 (6.7%)	0.81 (0.79, 0.82)	0.75	−6.07 (−69.81, 1.89)	0.15	-	-
Autoimmune diseases^#^	Relatives of controls	70 (1.8%)			Median = 24		Median = 1	
	1° relatives of cases	19 (2.2%)	1.01 (1.00, 1.01)	0.98	3.88 (3.05, 4.71)	0.37	−0.15 (−0.47, 0.17)	0.40
	2° relatives of cases	1 (0.5%)	0.48 (0.47, 0.49)	0.23	12.51 (9.28, 15.75)	0.43	-	-
	3° relatives of cases	1 (3.3%)	2.44 (2.38, 2.49)	0.22	−17.49 (−20.72, −14.25)	0.30	-	-
Infectious diseases^§^	Relatives of controls	1379 (36.5%)			Median = 4		Median = 1	
	1° relatives of cases	267 (31.2%)	0.95 (0.94, 0.95)	0.55	1.07 (−1.13, 1.29)	0.48	−0.09 (−0.24, 0.05)	0.21
	2° relatives of cases	60 (32.4%)	0.96 (0.96, 0.97)	0.83	1.24 (−1.14, 1.76)	0.23	−0.14 (−0.42, 0.14)	0.34
	3° relatives of cases	13 (43.3%)	1.41 (1.40, 1.43)	0.37	−3.01 (−8.15, −1.12)	0.03^†^	−0.34 (−0.99, 0.31)	0.30

Missing results indicate categories for which insufficient numbers were available for analysis.

* The CVD incidence outcome was adjusted for sex and age in 2008; ^§^ The asthma/allergy and infectious disease age at onset outcomes were adjusted for sex; ^#^ The autoimmune disease incidence outcome was adjusted for sex and age in 2008. The autoimmune disease number of hospital admissions per person outcome was adjusted for age in 2008; ^†^ *P*<0.05.

## Discussion

This is the first study using population-linked data to investigate the incidence of infection and immune-related diseases in KD patients and their relatives. We analysed the unique linked health data resources available in WA and used hospital admission as a marker of illness severity.[17] Patterns of infection and asthma/allergy differed significantly between KD cases and controls. Children with KD were more likely to be admitted to hospital for infectious diseases and asthma/allergy and had more infectious disease admissions per person than non-KD controls. The majority of these infectious and allergy/asthma admissions occurred prior to KD and are therefore unlikely to reflect immune dysfunction resulting from KD itself. Relatives of KD children also had more hospital admissions for asthma/allergy and for infection, compared with control relatives matched for degree of relatedness, although the effect sizes were smaller. Admission diagnoses of viral infections were more common, and skin and soft tissue infections less common in KD children and their relatives compared to control families. These data suggest that inherited immunological differences and/or differing rates of immune maturation[22] in KD patients may affect the epidemiologic patterns of other immune-related conditions.

The incidence and the total number of infectious disease admissions remained significantly increased in KD patients when the admissions in the two weeks prior to the KD diagnosis were excluded. Previous studies have shown that around the time of the KD diagnosis, children often have symptoms of other infectious diseases.[23] This partly reflects the diagnostic difficulties in KD, where the differential diagnosis is broad and there is no diagnostic test.[2] However there is also a high incidence of microbiologically confirmed concurrent infections at the time of KD diagnosis;[24] therefore exclusion of infectious admission in the two weeks prior to the KD diagnosis may be overly conservative and is likely to have underestimated the true effect size of infection-related admissions in the KD group. Viral infections, which were diagnosed clinically (the study period predates the introduction of rapid viral diagnostics), are amongst the most common concurrent infections at the time of KD diagnosis[24] and accounted for some of the overall increased infectious disease admissions in KD patients and their families. It is possible that the immunogenetic determinants of KD also alter the susceptibility to (or the severity of) viral infection. A viral trigger may be involved in initiating the abnormal immune response in KD.[25]

Children with KD were more likely to be admitted to hospital with allergy and asthma, with the majority of these admissions occurring before the KD admission. This suggests that a distinct immune phenotype may be associated with an increased risk of both KD and asthma/allergy. In acute KD there is sustained neutrophil activation, with increased release of human neutrophil elastase and matrix metalloproteinases,[26] and similar patterns may be important in childhood asthma.[27] Immunogenetic variation in the innate immune response may contribute to the shared risk of KD and infection and/or asthma/allergy. For example common functional variants in the gene for mannose binding lectin (MBL), a key complement-like constituent of innate immune defence, have been associated with susceptibility to KD,[28] and also coronary artery damage.[29] A role for MBL variants in susceptibility to certain infections, and to asthma and atopy has been reported, but data are inconsistent.[30] The available data suggest that KD is unlikely to be triggered by common allergens.[31]

The balance of T helper (Th) 1 and 2 responses is perturbed in acute KD,[32], [33], [34] and during the sub-acute phase there is a Th2 predominance.[35] Persistent immunological changes are also observed in the weeks following KD and persist for some months. These changes include a selective and prolonged T cell unresponsiveness to activation via the T cell antigen receptor CD3 and an incomplete responsiveness to measles-mumps-rubella vaccination.[22] A number of studies report increased asthma and allergy following KD, including an increased risk of atopic dermatitis suggested in US children.[36] A Japanese epidemiological survey reported that KD-affected children were almost twice as likely to develop dermatitis and/or allergic rhinitis and more likely to have a family history of allergy than non-KD controls.[37] Similar findings are reported from a sibling study from Singapore; KD-affected children had more allergy and asthma following KD than their non-KD affected siblings.[38] These data may also reflect the immunological consequences of KD, as well as an underlying susceptibility to both KD and asthma/allergy; the investigation of children following the KD illness does not differentiate between the two possibilities. In our study the majority of infectious and allergy/asthma admissions in KD patients occurred prior to the KD illness, suggesting that there may be underlying immunological differences in this population and that the increased admissions do not reflect perturbation of immune function due to the KD itself.

Inflammatory and autoimmune diseases show clustering in epidemiologic studies, with shared pathophysiology,[39] and increasing evidence of common genetic determinants.[40], [41] It has been suggested that environmental insults in early life may lead to broad patterns of immune dysfunction and clustering of conditions characterised by immune dysfunction.[42] Investigation of common pathogenic pathways may therefore be informative about KD and other inflammatory diseases where the aetiology and pathogenesis remain incompletely understood.

The strengths of our analysis include the novelty of the approach, the use of population data and the linked family data, which has been extensively validated in previous studies.[12] We excluded individuals that were born outside of WA, for whom childhood admission data were unavailable. As the diagnosis of KD is made largely on a constellation of non-specific clinical features that appear sequentially, the syndrome is often initially misdiagnosed as infectious and patients may be admitted to hospital more than once during the KD illness. We therefore excluded all admissions with infectious disease codings in the two weeks preceding the KD-related admission, to reduce likely inflation of infectious disease-related admissions in KD patients that were part of the KD diagnostic process.

We acknowledge some limitations to the study. The use of linked anonymised population data meant that the diagnosis of KD could not be confirmed on an individual case basis from the clinical notes. Previous studies involving KD patients in WA[9] indicate that the diagnosis of KD is made in accordance with the internationally accepted case definition (Burgner D, unpublished observations).[2] We are therefore confident that the KD coding is likely to be specific and any mis-diagnoses would not introduce systematic bias. Data on Aboriginal status were not readily available for the current analysis. Approximately 3.8% of the population of WA is Aboriginal, and Aboriginal children have a higher rate of hospitalisations for many infectious diseases than non-Aboriginal children.[17] Interestingly KD appears relatively rare in Aboriginal children; in the only published Australian KD epidemiology, only 2 of 139 children identified by active surveillance were Aboriginal.[43] The analyses are therefore unlikely to be biased by Aboriginal status. We used hospital admission as a proxy for disease severity, as previously,[17] because data for primary care and emergency department attendances were not available as part of the WADLS. Moreover, health-seeking behaviour differs between families and may not necessarily reflect illness severity. This may be an important consideration in families of children who have had KD, as the diagnosis results in considerable parental anxiety,[44] and medical consultations in ambulatory care may be increased. Analysis using more severe phenotypes is relatively conservative, but gives greater confidence in the validity of the findings. Clinical criteria for hospitalisation are likely to be uniform across WA pediatric centres. We are unable to comment on the epidemiologic patterns of less severe manifestations of immune-related illnesses, which account for the majority of pediatric morbidity. This is particularly true of mild infections and allergic conditions, as well as diseases such as psoriasis, which rarely require hospitalisation. Investigation of less severe, non-hospitalised illness would require prospective surveillance and is not amenable to population-based linkage analysis. Western Australia has a largely Caucasian population (detailed ethnicity data were not available for this analysis),[17] and the incidence of KD is therefore lower than in high incidence Asian countries. Further studies in high incidence populations are warranted to confirm the epidemiological patterns described. As the clinical phenotype and epidemiology of KD is consistent worldwide, it is plausible that similar patterns of co-morbidities will be present in other populations, reflecting shared underlying heritable mechanisms. Finally our study lacked power to comment with confidence on the incidence of cardiovascular disease in relatives of KD patients. As KD is suggested to predispose to accelerated atherosclerosis,[45], [46] and atherosclerosis-related outcomes may have genetic determinants,[47] this is clearly of interest. The low incidence of autoimmune diseases in children and their management without hospital admission limited meaningful analyses of these conditions.

In summary, this is the first description of patterns of hospital admission with common immune-related diseases in KD patients and their families. We have shown that KD-affected children have more admissions with infectious diseases and allergy/asthma, which may reflect common host determinants and pervasive differences in immune phenotype that result in differential disease risks.

## Supporting Information

Figure S1 Number of times KD cases (*n* = 295) and controls (*n* = 598) were admitted to hospital for infectious diseases. KD case admissions coded as infectious disease up to 2 weeks prior to KD diagnosis have been excluded.
(TIF)

Table S1 ICD codes and diagnostic groups by CCS.
(DOC)

Table S2 Infectious disease admissions by CCS diagnostic code in KD cases, controls, KD case relatives and control relatives.
(DOC)

Table S3 Asthma/allergy admissions by CCS diagnostic code in KD cases and controls.
(DOC)
