# Supplementary figures and images for: Hospitalisation with Infection, Asthma and Allergy in Kawasaki Disease Patients and Their Families: Genealogical Analysis Using Linked Population Data
Source: PLoS One. 2011 Nov 28;6(11):e28004. doi: 10.1371/journal.pone.0028004 (PMC3225371; doi:10.1371/journal.pone.0028004)

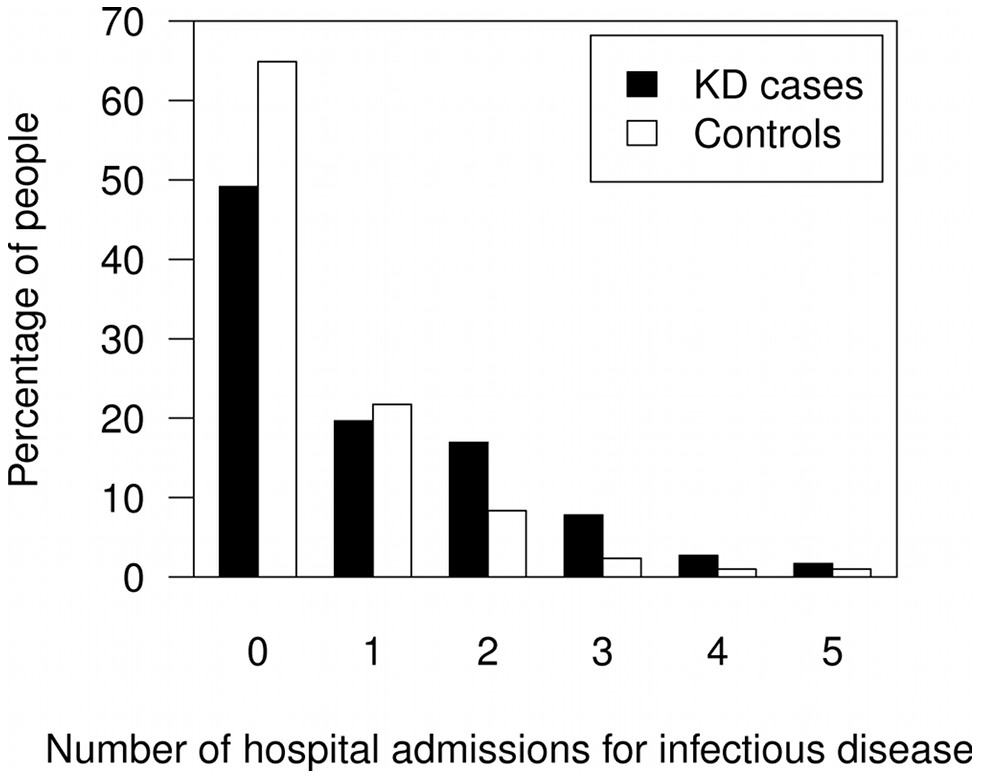

Supplement: Figure S1 — Number of times KD cases (n = 295) and controls (n = 598) were admitted to hospital for infectious diseases. KD case admissions coded as infectious disease up to 2 weeks prior to KD diagnosis have been excluded. (TIF) [file pone.0028004.s001.tif]
