# Supplementary material for: Hospitalisation with Infection, Asthma and Allergy in Kawasaki Disease Patients and Their Families: Genealogical Analysis Using Linked Population Data
Source: PLoS One. 2011 Nov 28;6(11):e28004. doi: 10.1371/journal.pone.0028004 (PMC3225371; doi:10.1371/journal.pone.0028004)
Supplement: Table S1 — ICD codes and diagnostic groups by CCS. (DOC) [file pone.0028004.s002.doc]

| Diagnostic disease group | CCS codes | ICD10.5 codes |
| --- | --- | --- |
| Cardiovascular disease | 53, 100, 101, 104, 109, 110, 111, 114, 115, 117, 259 | E78.0, E78.1, E78.2, E78.3, E78.4, I20.0, I20.1, I20.8, I20.9, I21.0, I21.1, I21.2, I21.3, I21.4, I24.0, I24.1, I24.8, I25.10, I25.11, I25.13, I25.2, I25.4, I25.8, I25.9, I60.9, I61.9, I62.0, I62.1, I62.9, I63.0, I63.2, I63.3, I63.4, I63.5, I64, I65.0, I65.1, I65.2, I65.3, I65.8, I65.9, I66.8, I66.9, I67.2, I70.0, I70.1, I70.20, I70.21, I70.22, I70.23, I70.8, I70.9, I71.00, I71.01, I71.02, I71.03, I71.1, I71.2, I71.3, I71.4, I71.5, I71.6, I71.8, I71.9, I72.0, I72.1, I72.2, I72.3, I72.4, I72.8, I72.9, I73.9, K55.0, K55.1, K55.9, Z95.1, Z95.5 |
| Asthma/allergy | 127, 128, 134, 200, 204, 253, 259 | J30.3, J30.4, J34.3, J44.8, J45.0, J45.1, J45.9, J46, L20.8, L22, L23.0, L23.2, L23.3, L23.5, L23.6, L23.7, L23.8, L23.9, L24.0, L24.1, L24.2, L27.0, L27.2, L27.8, L27.9, L50.0, L50.1, L50.2, L50.3, L50.4, L50.5, L50.8, L50.9, L55.8, L56.9, L57.1, L57.8, L59.8, M13.81, M13.82, M13.83, M13.84, M13.85, M13.86, M13.87, M13.88, M13.89, T78.0, T78.2, T78.4, Z51.6, Z88.6, Z88.7, Z88.8 |
| Infectious diseases | 1, 2, 3, 4, 6, 7, 8, 9, 76, 77, 78, 88, 90, 91, 92, 93, 95, 96, 97, 122, 123, 124, 125, 126, 133, 134, 135, 139, 148, 151, 152, 155, 159, 168, 197, 201, 211, 212, 224, 247 | A00.0, A00.1, A00.9, A01.0, A01.1, A01.2, A01.3, A01.4, A02.0, A02.1, A02.2+, A02.8, A02.9, A03.0, A03.1, A03.2, A03.3, A03.8, A03.9, A04.0, A04.1, A04.2, A04.3, A04.4, A04.5, A04.6, A04.7, A04.8, A04.9, A05.0, A05.1, A05.2, A05.3, A05.8, A05.9, A06.0, A06.1, A06.2, A06.4, A06.5+, A06.6+, A06.7, A06.8, A06.9, A07.0, A07.1, A07.2, A07.3, A07.8, A07.9, A08.0, A08.1, A08.2, A08.3, A08.5, A09, A15.0, A15.1, A15.2, A15.3, A15.4, A15.5, A15.6, A15.7, A15.8, A16.0, A16.1, A16.2, A16.3,A16.4, A16.5, A16.7, A16.8, A17.0+, A17.1+, A17.8+, A17.9+, A18.0+, A18.1, A18.1+, A18.2, A18.3+, A18.4, A18.5+, A18.6+, A18.7+, A18.8+, A19.2, A19.8, A19.9, A20.0, A20.1, A20.2, A20.7, A20.8, A20.9, A21.0, A21.1, A21.2, A21.3, A21.8, A21.9, A22.0, A22.1, A22.2, A22.7, A22.8, A22.9, A23.0, A23.1, A23.2, A23.3, A23.8, A23.9, A24.0, A24.4, A25.0, A25.1, A25.9, A26.9, A27.0, A27.8, A27.9, A28.0, A28.1, A28.8, A28.9, A30.0, A30.1, A30.3, A30.5, A30.8, A30.9, A31.0, A31.1, A32.9, A35, A36.0, A36.1, A36.2, A36.3, A36.8, A36.8+, A36.9, A37.0, A37.1, A37.8, A37.9, A38, A39.0+, A39.1+, A39.4, A39.5+, A39.8, A39.8+, A39.9, A40.3, A40.9, A41.2, A41.3, A41.4, A41.51, A41.52, A41.58, A41.8, A41.9, A42.0, A42.1, A42.2, A42.8, A42.9, A44.9, A46, A48.0, A48.8, A49.9, A63.0, A65, A66.0, A66.1, A66.2, A66.3, A66.4, A66.5, A66.6, A66.7, A66.8, A66.9, A67.0, A67.1, A67.2, A67.3, A67.9, A68.0, A68.1, A68.9, A69.1, A69.2, A69.8, A69.9, A70, A70+, A71.0, A71.1, A71.9, A74.0+, A74.8, A74.9, A75.0, A75.1, A75.2, A75.3, A75.9, A77.0, A77.1, A77.2, A77.3, A77.8, A77.9, A78, A79.0, A79.1, A79.8, A79.9, A80.3, A80.4, A80.9, A81.0, A81.1, A81.2, A81.8, A81.9, A82.9, A83.0, A83.1, A83.2, A83.3, A83.4, A83.5, A83.8, A83.9, A84.0, A84.1, A84.8, A84.9, A85.2, A87.0+, A87.1+, A87.2, A88.8, A89, A90, A92.2, A92.8, A93.1, A93.2, A93.8, A94, A95.0, A95.1, A95.9, A96.8, A98.0, A98.1, A98.2, A98.5, B00.0, B00.3+, B00.4+, B00.5+, B00.7, B01.1+, B01.2+, B01.8, B01.9, B02.1+, B02.2+, B02.3+, B02.8, B02.9, B03, B05.0+, B05.2+, B05.8, B05.9, B06.0+, B06.8, B06.9, B07, B08.0, B08.1, B08.3, B08.4, B08.5, B08.8, B09, B25.9, B26.0+, B26.1+, B26.2+, B26.3+, B26.8, B26.8+, B26.9, B27.9, B30.0+, B30.1+, B30.2+, B30.3+, B30.8+, B33.0, B33.2, B33.8, B34.0, B34.1, B34.8, B34.9, B35.0, B35.1, B35.2, B35.3, B35.4, B35.6, B35.8, B35.9, B36.0, B36.1, B36.2, B36.3, B36.8, B36.9, B37.0, B37.1, B37.2, B37.3+, B37.4+, B37.5+, B37.6+, B37.81, B37.88, B37.9, B38.0, B38.1, B38.2, B38.3, B38.4+, B38.8, B38.9, B39.2, B39.4, B39.5, B39.9, B40.9, B41.9, B42.0+, B43.9, B44.9, B45.9, B46.9, B47.0, B47.9, B48.0, B48.1, B48.2, B48.7, B48.8, B50.8, B50.9, B51.9, B52.9, B53.0, B53.8, B54, B55.0, B55.1, B55.2, B55.9, B56.0, B56.1, B56.9, B57.1, B57.2, B57.5, B58.0+, B58.1+, B58.2+, B58.3+, B58.8, B58.9, B59, B60.0, B65.0, B65.1, B65.2, B65.3, B65.8, B65.9, B66.0, B66.1, B66.3, B66.4, B66.5, B66.8, B66.9, B67.0, B67.1, B67.3, B67.3+, B67.4, B67.5, B67.6, B67.7, B67.8, B67.9, B68.0, B68.1, B68.9, B69.9, B70.0, B70.1, B71.0, B71.8, B71.9, B72, B73, B74.0, B74.1, B74.3, B74.4, B74.8, B74.9, B75, B76.0, B76.1, B77.9, B78.9, B79, B80, B81.0, B81.1, B81.2, B81.4, B81.8, B82.0, B82.9, B83.0, B83.1, B83.8, B83.9, B85.0, B86, B87.9, B88.0, B88.2, B88.3, B88.8, B88.9, B89, B90.0, B90.1, B90.2, B90.8, B90.9, B91, B94.0, B94.1, B94.8, B95.0, B95.1, B95.2, B95.41, B95.42, B95.48, B95.6, B95.7, B95.8, B96.2, B96.3, B96.4, B96.5, B96.6, B96.7, B96.81, B96.88, G00.0, G00.1, G00.1*, G00.2, G00.3, G00.8, G00.9, G02.0*, G02.1*, G02.8*, G03.0, G03.1, G03.9, G04.0, G04.8, G04.9, G05.1*, G05.2*, G06.0, G06.1, G06.2, G09, G61.0, G92, H00.0, H01.0, H01.1, H01.8, H01.9, H03.0*, H03.1*, H04.0, H04.3, H04.4, H05.0, H05.1, H06.1*, H10.0, H10.1, H10.2, H10.3, H10.4, H10.5, H10.8, H10.9, H13.1*, H13.2*, H15.0, H15.1, H15.8, H16.1, H16.2, H16.3, H16.8, H16.9, H19.2*, H19.3*, H20.0, H20.1, H20.2, H20.8, H20.9, H30.0, H30.1, H30.2, H30.8, H30.9, H40.4, H44.0, H44.1, H46, H70.0, H82, I00, I01.0, I01.1, I01.2, I01.8, I01.9, I02.0, I09.0, I09.2, I09.8, I09.9, I30.0, I30.8, I30.9, I32.8*, I33.0, I33.9, I39.8*, I40.0, I40.1, I40.8, I40.9, I41.8*, J00, J01.0, J01.1, J01.2, J01.3, J01.8, J01.9, J02.0, J02.9, J03.9, J04.0, J04.1, J04.2, J05.0, J05.1, J06.0, J06.8, J06.9, J10.0, J10.1, J10.8, J12.0, J12.1, J12.2, J12.8, J12.9, J13, J14, J15.0, J15.1, J15.2, J15.3, J15.4, J15.5, J15.6, J15.7, J15.8, J15.9, J16.8, J17.0*, J17.1*, J17.2*, J17.8*, J18.0, J18.8, J20.9, J21.9, J32.0, J32.1, J32.2, J32.3, J32.8, J32.9, J35.0, J35.1, J35.2, J35.3, J35.8, J35.9, J36, J39.0, J39.1, J85.2, J99.0*, K63.0, K65.0, K65.8, K65.9, K67.8*, K75.0, K75.1, K90.8+, L01.0, L02.0, L02.1, L02.2, L02.3, L02.4, L02.8, L02.9, L03.01, L03.02, L03.10, L03.11, L03.2, L03.3, L03.8, L03.9, L04.9, L05.0, L05.9, L08.0, L08.8, L08.9, L98.0, M00.90, M00.91, M00.92, M00.93, M00.94, M00.95, M00.96, M00.97, M00.98, M00.99, M01.30*, M01.31*, M01.32*, M01.33*, M01.34*, M01.35*, M01.36*, M01.37*, M01.38*, M01.39*, M01.50*, M01.51*, M01.52*, M01.53*, M01.54*, M01.55*, M01.56*, M01.57*, M01.58*, M01.59*, M01.60*, M01.61*, M01.62*, M01.63*, M01.64*, M01.65*, M01.66, M01.67*, M01.68, M01.69*, M01.80*, M01.81*, M01.82*, M01.83, M01.83*, M01.84*, M01.85*, M01.86*, M01.87*, M01.88*, M01.89*, M02.11, M02.12, M02.13, M02.14, M02.15, M02.16, M02.17, M02.18, M02.19, M02.30, M02.31, M02.32, M02.33, M02.34, M02.35, M02.36, M02.37, M02.38, M02.39, M35.2, M60.09, M86.10, M86.11, M86.12, M86.13, M86.14, M86.15, M86.16, M86.17, M86.18, M86.19, M86.60, M86.67, M86.68, M86.69, M86.90, M86.91, M86.92, M86.93, M86.94, M86.95, M86.96, M86.97, M86.98, M86.99, M89.60, M89.61, M89.62, M89.63, M89.64, M89.65, M89.66, M89.67, M89.68, M89.69, M90.20*, M90.21*, M90.22*, M90.23*, M90.24*, M90.25*, M90.26*, M90.27*, M90.28*, M90.29*, N39.0, N72, P23.9, P37.5, P38, P39.0, P39.1, P39.8, R09.1, Z86.10, Z86.11, Z86.12, Z86.13, Z86.18 |
| Autoimmune diseases | 48, 49, 50, 59, 80, 95, 117, 134, 151, 155, 186, 198, 200, 202, 210, 211 | D59.1, E03.2, E03.8, E03.9, E04.0, E04.1, E04.2, E04.9, E05.0, E05.1, E05.2, E05.3, E05.8, E05.9, E06.0, E06.1, E06.3, E06.5, E06.9, E07.0, E07.1, E07.8, E07.9, E10.00, E10.10, E10.20+, E10.21+, E10.30+, E10.31+, E10.40+, E10.41+, E10.50, E10.60, E10.80, E10.90, E11.10, E89.0, G35, G70.0, J31.0, J34.3, K74.5, K90.0, L40.5+, L94.0, M05.09, M05.19+, M05.39+, M06.49, M06.99, M08.09, M08.49, M12.09, M30.0, M32.9, M33.1, M33.2, M35.0, M35.3, M35.8, M35.9, M45.09, O24.3, R94.6 |
| Crohn disease and ulcerative colitis | 144 | K50.0, K50.1, K50.8, K50.9, K51.0, K51.1, K51.2, K51.3, K51.4, K51.8, K51.9 |
| Psoriasis | 198 | L40.5+, L40.8 |

**Supplementary Table 1: ICD codes and diagnostic groups grouped by Clinical Classification Software**
