# Supplementary material for: Hospitalisation with Infection, Asthma and Allergy in Kawasaki Disease Patients and Their Families: Genealogical Analysis Using Linked Population Data
Source: PLoS One. 2011 Nov 28;6(11):e28004. doi: 10.1371/journal.pone.0028004 (PMC3225371; doi:10.1371/journal.pone.0028004)
Supplement: Table S2 — Infectious disease admissions by CCS diagnostic code in KD cases, controls, KD case relatives and control relatives. (DOC) [file pone.0028004.s003.doc]

| **CCS Code** | **CCS Diagnostic Category** | **KD Cases** | | **Controls** | | **KD Case Relatives** | | **Control Relatives** | |
| --- | --- | --- | --- | --- | --- | --- | --- | --- | --- |
|  |  | n | % | n | % | n | % | n | % |
| 126 | Other upper respiratory infections | 63 | 14.5% | 77 | 16.6% | 181 | 14.2% | 484 | 13.1% |
| 7 | Viral infection | 61 | 14.1% | 35 | 7.6% | 145 | 11.3% | 283 | 7.7% |
| 124 | Acute and chronic tonsillitis | 61 | 14.1% | 82 | 17.7% | 178 | 13.9% | 366 | 9.9% |
| 135 | Intestinal infection | 51 | 11.8% | 47 | 10.2% | 127 | 9.9% | 492 | 13.4% |
| 3 | Bacterial infection; unspecified site | 41 | 9.4% | 51 | 11.0% | 169 | 13.2% | 462 | 12.5% |
| 90 | Inflammation; infection of eye (except that caused by tuberculosis or sexually transmitted disease) | 31 | 7.1% | 15 | 3.2% | 32 | 2.5% | 114 | 3.1% |
| 125 | Acute bronchitis | 24 | 5.5% | 30 | 6.5% | 60 | 4.7% | 221 | 6.0% |
| 197 | Skin and subcutaneous tissue infections | 21 | 4.8% | 35 | 7.6% | 100 | 7.8% | 382 | 10.4% |
| 122 | Pneumonia (except that caused by tuberculosis or sexually transmitted disease) | 19 | 4.4% | 15 | 3.2% | 72 | 5.6% | 240 | 6.5% |
| 224 | Other perinatal conditions | 18 | 4.1% | 33 | 7.1% | 43 | 3.4% | 90 | 2.4% |
| 4 | Mycoses | 12 | 2.8% | 9 | 1.9% | 21 | 1.6% | 63 | 1.7% |
| 201 | Infective arthritis and osteomyelitis (except that caused by tuberculosis or sexually transmitted disease) | 8 | 1.8% |  |  | 9 | 0.7% | 23 | 0.6% |
| 159 | Urinary tract infections | 8 | 1.8% | 13 | 2.8% | 56 | 4.4% | 190 | 5.2% |
| 8 | Other infections; including parasitic | 5 | 1.2% | 12 | 2.6% | 21 | 1.6% | 125 | 3.4% |
| 123 | Influenza | 4 | 0.9% | 2 | 0.4% | 7 | 0.5% | 31 | 0.8% |
| 2 | Septicaemia (except in labour) | 3 | 0.7% | 1 | 0.2% | 7 | 0.5% | 34 | 0.9% |
| 76 | Meningitis (except that caused by tuberculosis or sexually transmitted disease) | 1 | 0.2% | 2 | 0.4% | 13 | 1.0% | 12 | 0.3% |
| 77 | Encephalitis (except that caused by tuberculosis or sexually transmitted disease) | 1 | 0.2% |  |  | 1 | 0.1% | 4 | 0.1% |
| 134 | Other upper respiratory disease | 1 | 0.2% |  |  | 1 | 0.1% |  |  |
| 247 | Lymphadenitis | 1 | 0.2% | 1 | 0.2% | 3 | 0.2% | 3 | 0.1% |
| 9 | Sexually transmitted infections (not HIV or hepatitis) |  |  | 1 | 0.2% | 1 | 0.1% | 1 | 0.0% |
| 92 | Otitis media and related conditions |  |  | 1 | 0.2% |  |  |  |  |
| 148 | Peritonitis and intestinal abscess |  |  | 1 | 0.2% | 8 | 0.6% | 6 | 0.2% |
| 168 | Inflammatory diseases of female pelvic organs |  |  |  |  | 16 | 1.3% | 34 | 0.9% |
| 130 | Pleurisy; pneumothorax; pulmonary collapse |  |  |  |  | 6 | 0.5% | 4 | 0.1% |
| 78 | Other CNS infection and poliomyelitis |  |  |  |  | 1 | 0.1% | 2 | 0.1% |
| 259 | Residual codes; unclassified |  |  |  |  | 1 | 0.1% | 3 | 0.1% |
| 97 | Peri-; endo-; and myocarditis; cardiomyopathy (except that caused by tuberculosis or sexually transmitted disease) |  |  |  |  |  |  | 7 | 0.2% |
| 95 | Other nervous system disorders |  |  |  |  |  |  | 5 | 0.1% |
| 1 | Tuberculosis |  |  |  |  |  |  | 2 | 0.1% |
| 133 | Other lower respiratory disease |  |  |  |  |  |  | 2 | 0.1% |
|  |  | 434 | 100% | 463 | 100% | 1279 | 100% | 3685 | 100% |

**Supplementary Table 2: Infectious disease admissions by Clinical Classification Software (CCS) diagnostic code in Kawasaki disease (KD) cases, controls, KD case relatives and control relatives.**
