# Supplementary material for: Hospitalisation with Infection, Asthma and Allergy in Kawasaki Disease Patients and Their Families: Genealogical Analysis Using Linked Population Data
Source: PLoS One. 2011 Nov 28;6(11):e28004. doi: 10.1371/journal.pone.0028004 (PMC3225371; doi:10.1371/journal.pone.0028004)
Supplement: Table S3 — Asthma/allergy admissions by CCS diagnostic code in KD cases and controls. (DOC) [file pone.0028004.s004.doc]

**Supplementary Table 3: Asthma/allergy admissions by Clinical Classification Software (CCS) diagnostic code in Kawasaki disease (KD) cases and controls.**
